# Supplementary material for: Molecular basis for allosteric agonism and G protein subtype selectivity of galanin receptors
Source: Nat Commun. 2022 Mar 15;13:1364. doi: 10.1038/s41467-022-29072-3 (PMC8924211; doi:10.1038/s41467-022-29072-3)
Supplement: Supplementary file 1 — Supplementary Information [file 41467_2022_29072_MOESM1_ESM.pdf]

Supplementary Information

Molecular basis for allosteric agonism and G protein subtype selectivity of galanin receptors

Supplementary Figure 1. Purification of galanin-GAL1R-G_i and galanin-GAL2R-G_q complexes and cryo-EM data processing

Supplementary Figure 2. Overall resolution analysis of electron density of transmembrane helices, helix 8, and galanin

Supplementary Figure 3. Sequence alignment of galanin and related peptides

Supplementary Figure 4. 2D presentation of interactions between galanin and galanin receptors

Supplementary Figure 5. Binding and function of galanin mutants on galanin receptors

Supplementary Figure 6. Saturation binding curves of galanin-A2 on GAL1R mutants

Supplementary Figure 7. Saturation binding curves of galanin-A2 on GAL2R mutants

Supplementary Figure 8. Galanin response curves on WT and mutant galanin receptors

Supplementary Figure 9. Spexin response curves on WT and mutant galanin receptors

Supplementary Figure 10. Detailed interactions between α5 helix of the Gα subunit and residues in cytoplasmic cavity of galanin receptors

Supplementary Table 1. Cryo-EM data collection, model refinement, and validation statistics

Supplementary Table 2. Binding and function of galanin mutants on WT galanin receptors

Supplementary Table 3. Binding of galanin-A2 and Function of galanin on GAL1R mutants

Supplementary Table 4. Binding of galanin-A2 and Function of galanin on GAL2R mutants

Supplementary Table 5. Function of spexin on GAL2R mutants

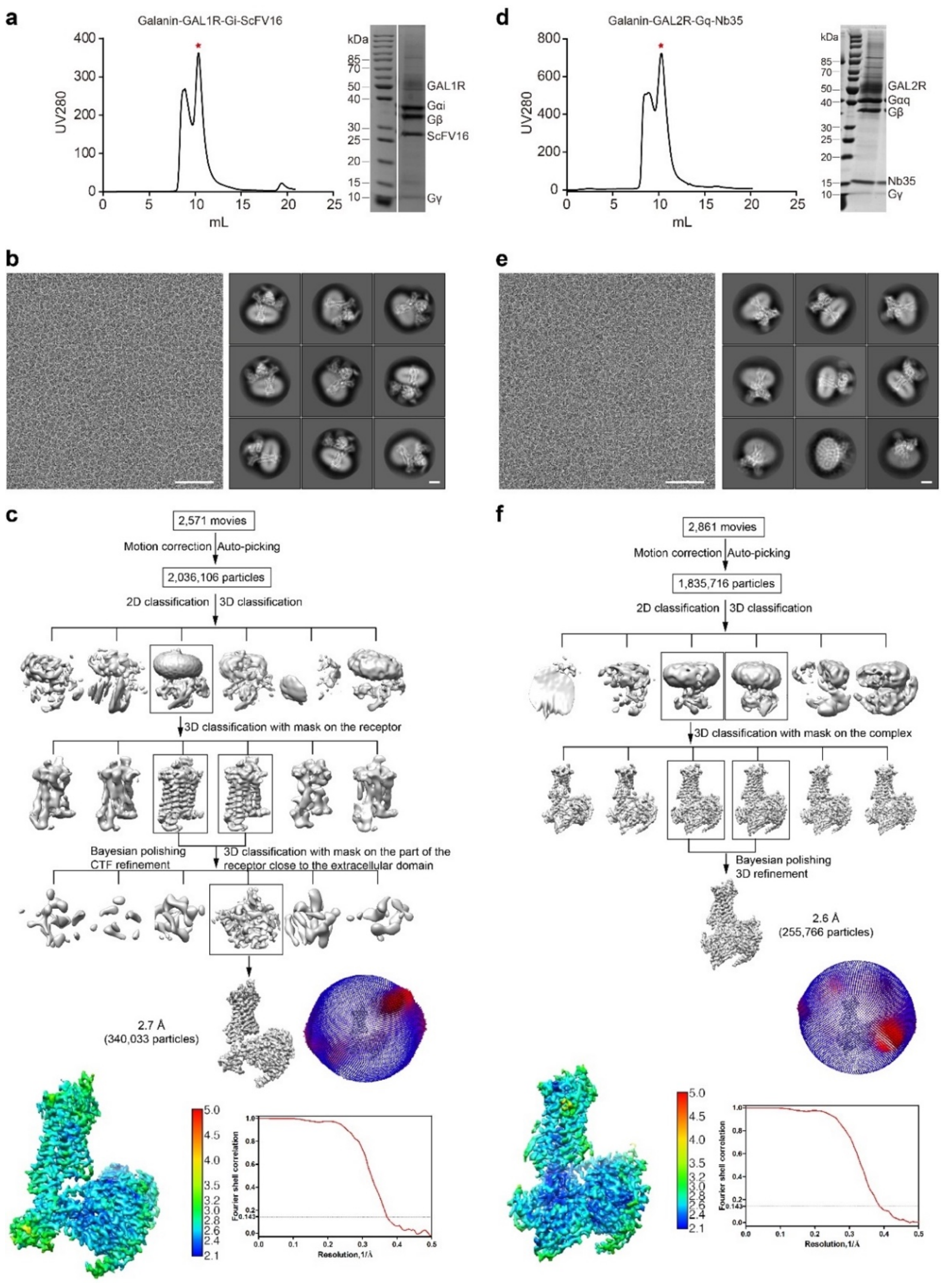

Supplementary Figure 1│Purification of galanin-GAL1R-G_i and galanin-GAL2R-G_q complexes and cryo-EM data processing. a, d, Representative elution profile and SDS-PAGE analysis of the galanin-GAL1R-G_i-scFv16 (**a**) and galanin-GAL2R-G_q-Nb35 complexes (**d**). Red asterisks denote the monomer of two complexes. The data collection was performed once. **b, e,** Cryo-EM micrographs of the galanin-GAL1R-G_i-scFv16 (**b**) and galanin-GAL2R-G_q-Nb35 complexes (**e**) (scale bar: 50 nm) and 2D class averages (scale bar: 5 nm). **c, f,** Flow chart of the cryo-EM data processing for the galanin-GAL1R-G_i-scFv16 (**c**) and galanin-GAL2R-G_q-Nb35 complexes (**f**).

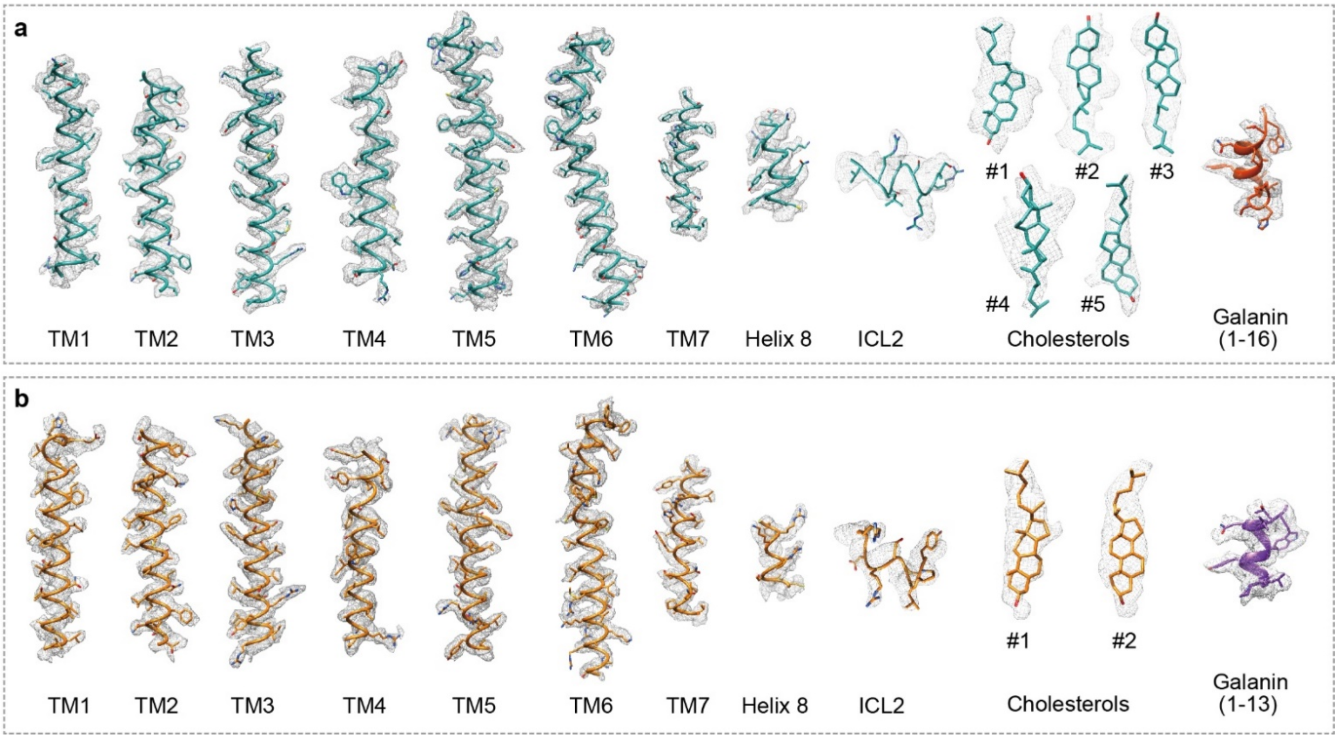

Supplementary Figure 2 | Overall resolution analysis of electron density of transmembrane helices, helix 8, and galanin. EM density and model of galanin-GAL1R-G_i (**a**) and galanin-GAL2R-G_q complexes (**b**).

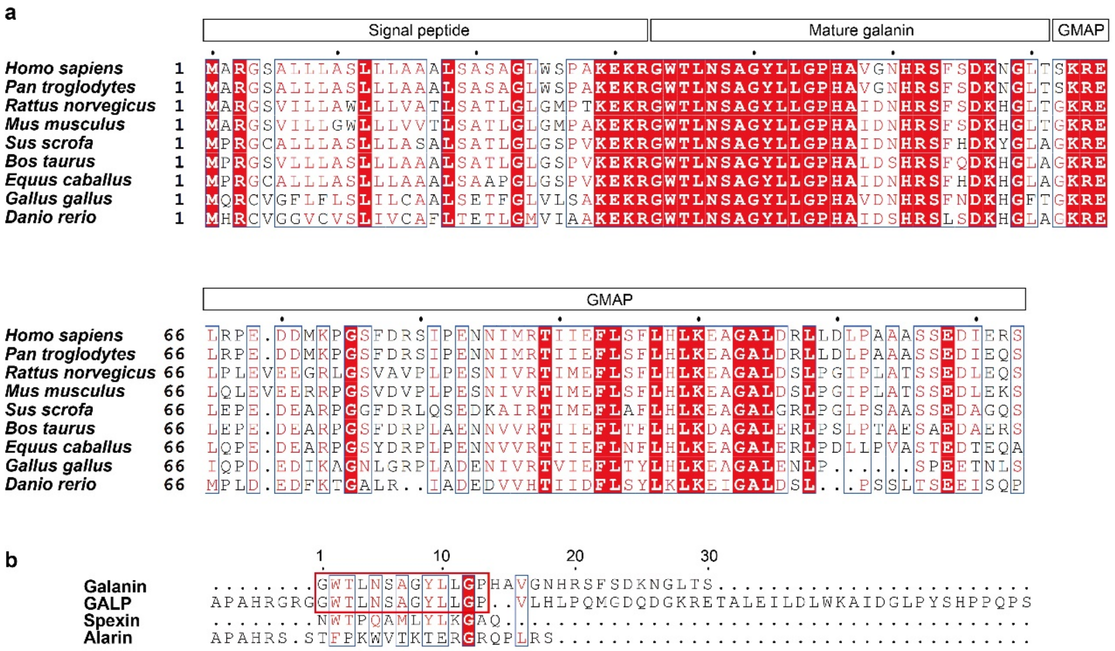

Supplementary Figure 3 | Sequence alignment of galanin and related peptides. a, Sequence alignment of galanin from different species. Signal peptide, mature galanin peptide, and galanin message-associated protein (GMAP) were labeled. **b,** Sequence alignment of galanin and related peptides, including galanin-related peptide (GALP), spexin, and alarin. The conserved 13 amino acids between galanin and GALP are highlighted in a red rectangle.

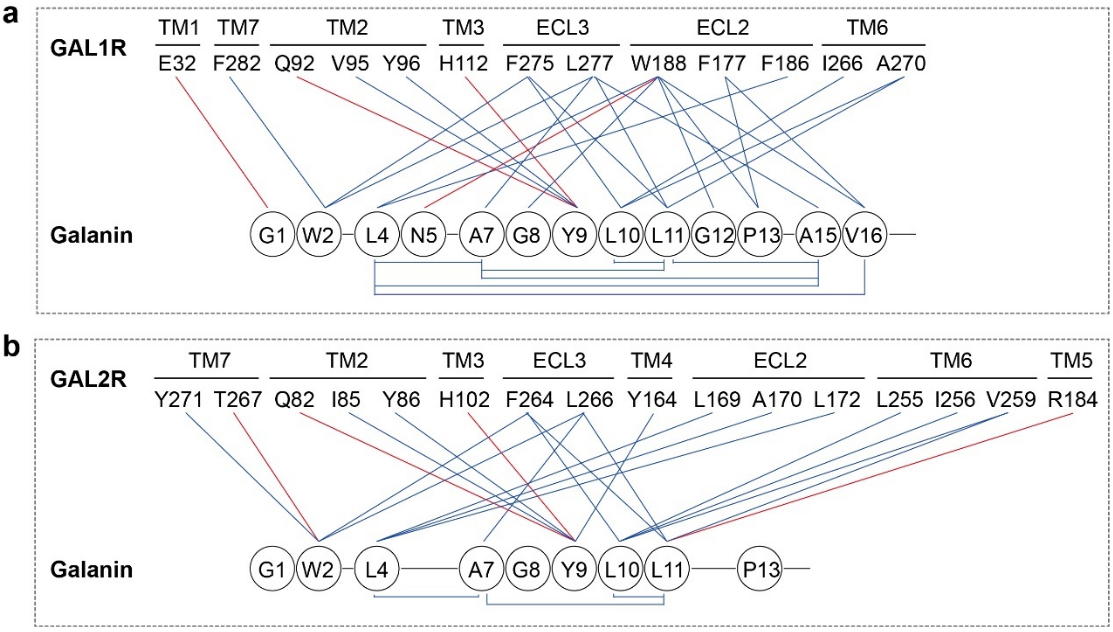

Supplementary Figure 4 | 2D presentation of interactions between galanin and galanin receptors. Detailed interactions between galanin and GAL1R (**a**) and GAL2R (**b**). Polar and hydrophobic interactions are shown as red and blue lines, respectively.

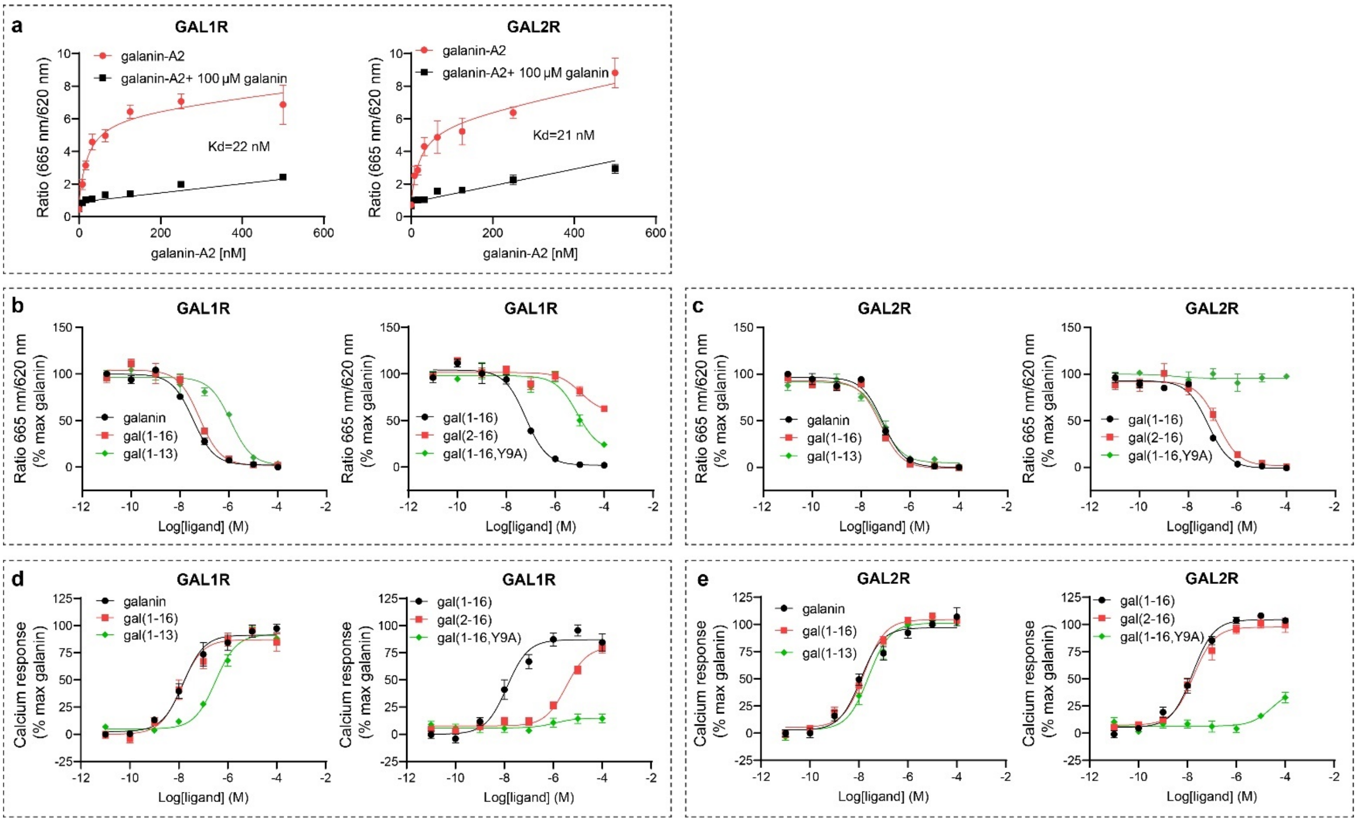

Supplementary Figure 5 | Binding and function of galanin mutants on galanin receptors. **a,** Saturation binding curves of galanin-A2 on GAL1R and GAL2R. **b, c,** Competition binding curves (galanin-A2 was used at 50 nM) of galanin mutants on GAL1R (**b**) and GAL2R (**c**). Both the saturation and competition binding assays were performed on HEK293 cells transfected with WT GAL1R or GAL2R. **d, e,** Calcium response curves of galanin mutants on GAL1R (**d**) and GAL2R (**e**). The WT receptors were transfected into HEK293/Gα_{16} cells and intracellular calcium signals were measured to reflect the activity of galanin mutants. Each point represents mean ± S.E.M. from at least three independent experiments (n = 4, **a**, and n = 6, **b-e**). Source data are provided as a Source Data file.

a

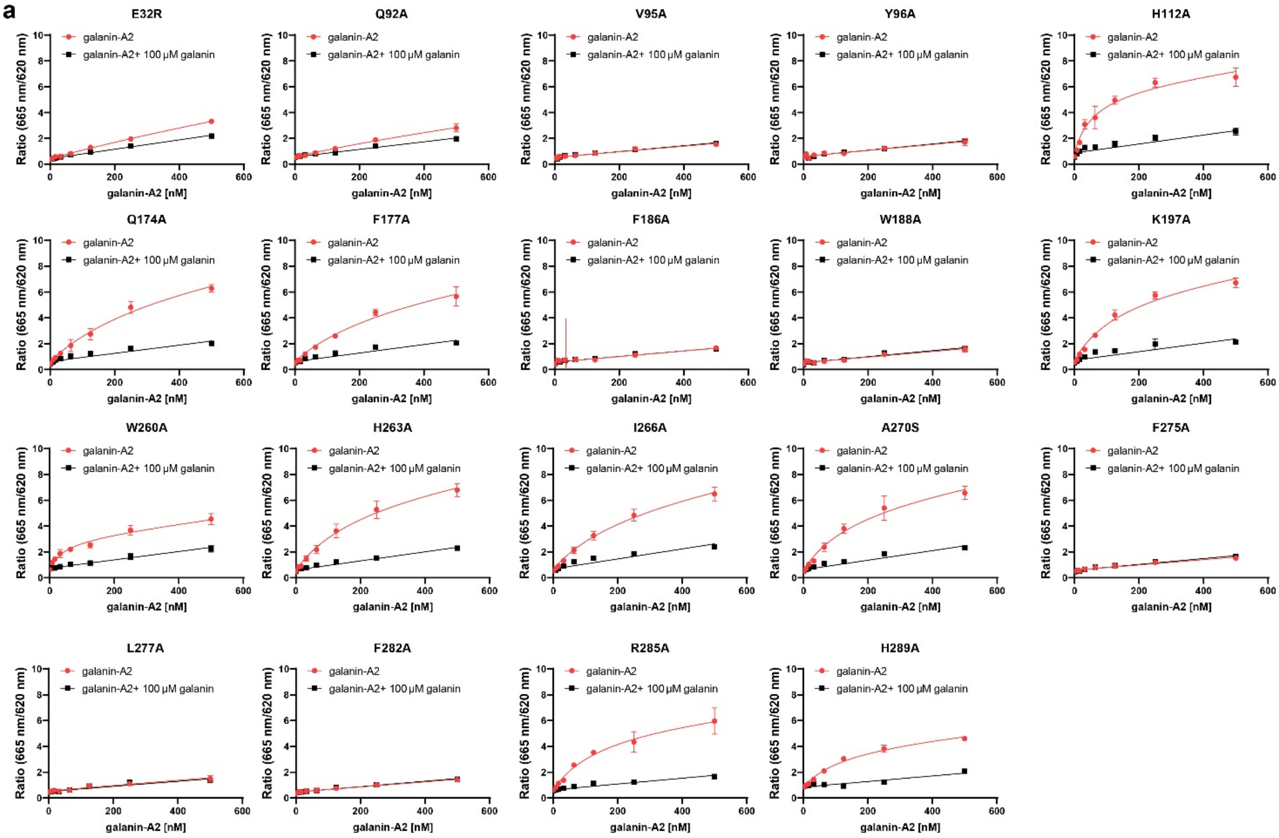

b

TM1	TM2			TM3	TM4	ECL2			TM5	TM6				ECL3		TM7		
1.31	2.61	2.64	2.65	3.29	4.64				5.35	6.48	6.51	6.54	6.58			7.32	7.35	7.39
**E32**	**Q92**	**V95**	**Y96**	**H112**	**Q174**	**F177**	**F186**	**W188**	**K197**	**W260**	**H263**	**I266**	**A270**	**F275**	**L277**	**F282**	**R285**	**H289**

Supplementary Figure 6 | Saturation binding curves of galanin-A2 on GAL1R mutants. **a,** Saturation binding experiments were performed on HEK293 cells transfected with different GAL1R mutants. Residues within 4 Å were tested. Each point represents mean ± S.E.M. from four independent experiments (n = 4). Source data are provided as a Source Data file. The Saturation binding curve of galanin-A2 on wild-type GAL1R is presented in Extended Data Fig. 5a. **b,** Location of GAL1R mutants.

a

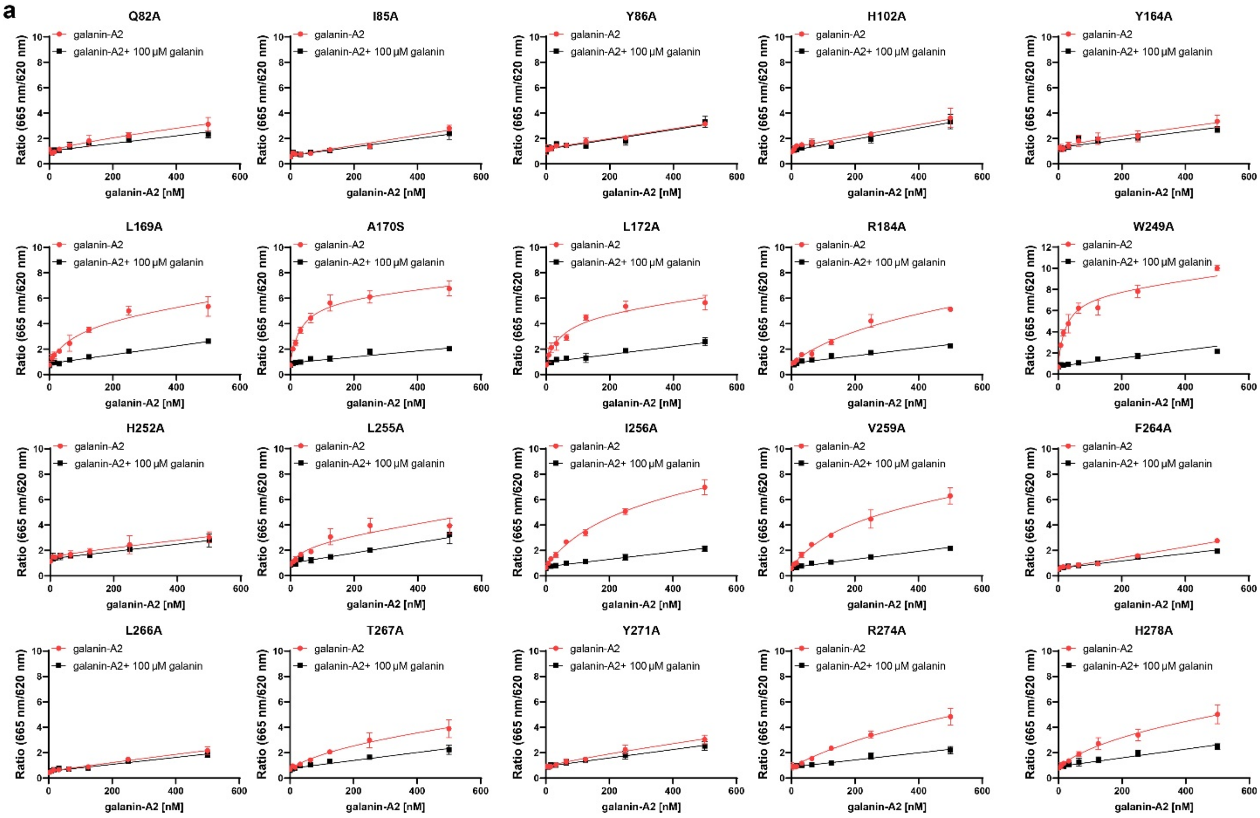

b

TM2			TM3	TM4	ECL2			TM5	TM6					ECL3		TM7			
2.61	2.64	2.65	3.29	4.64				5.35	6.48	6.51	6.54	6.55	6.58			7.28	7.32	7.35	7.39
**Q82**	**I85**	**Y86**	**H102**	**Y164**	**L169**	**A170**	**L172**	**R184**	**W249**	**H252**	**L255**	**I256**	**V259**	**F264**	**L266**	**T267**	**Y271**	**R274**	**R285**

Supplementary Figure 7 | Saturation binding curves of galanin-A2 on GAL2R mutants. **a,** Saturation binding experiments were performed on HEK293 cells transfected with different GAL2R mutants. Residues within 4 Å were tested. Each point represents mean ± S.E.M. from four independent experiments (n = 4). Source data are provided as a Source Data file. The Saturation binding curve of galanin-A2 on wild-type GAL2R is presented in Extended Data Fig. 5a. **b,** Location of GAL2R mutants.

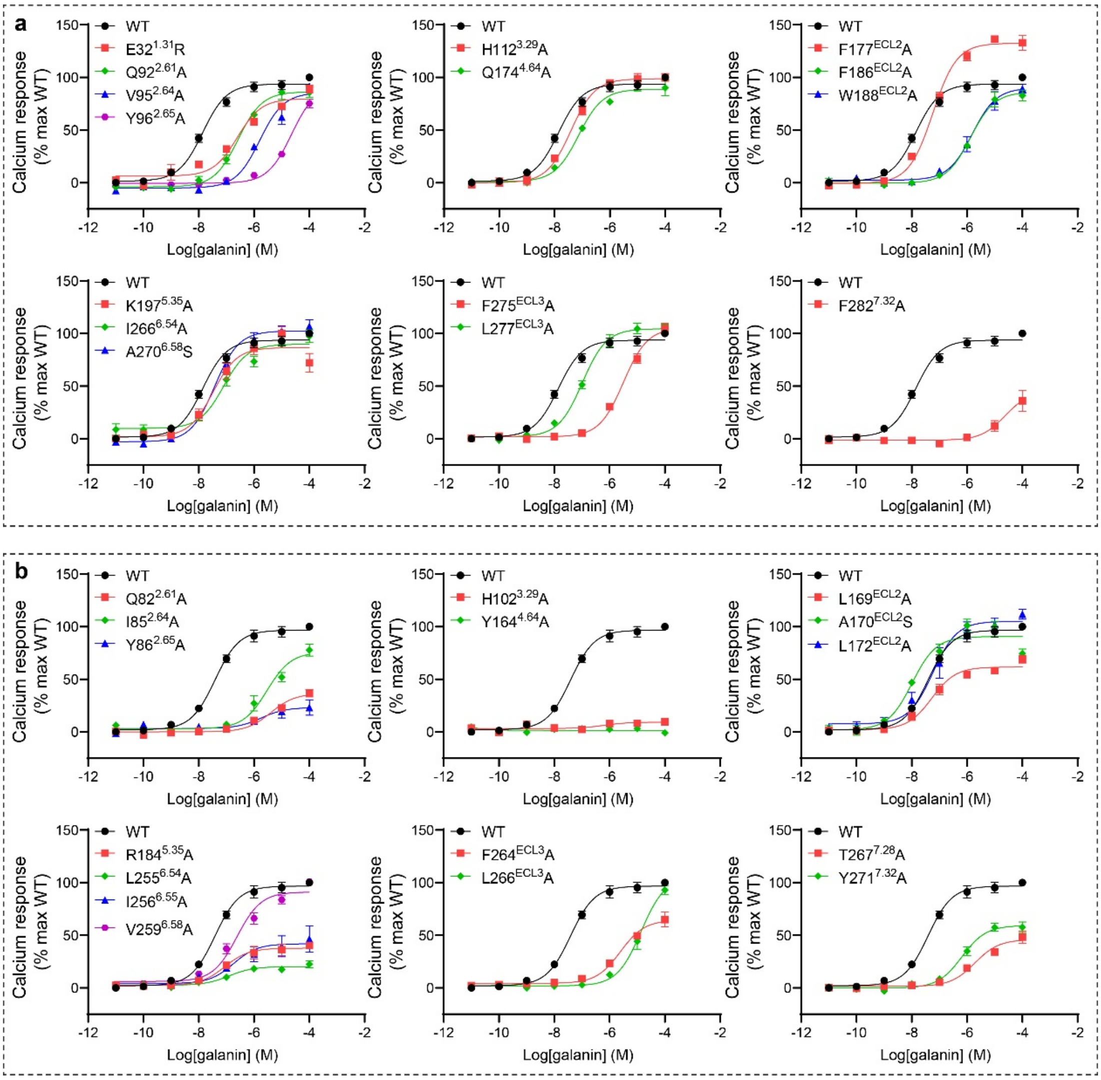

Supplementary Figure 8 | Galanin response curves on WT and mutant galanin receptors. WT or mutant GAL1R (**a**) and GAL2R (**b**) were transfected into HEK293/Gα_{16} cells and intracellular calcium signals were measured to reflect the activity of galanin. Each point represents mean ± S.E.M. from six independent experiments (n = 6). Source data are provided as a Source Data file.

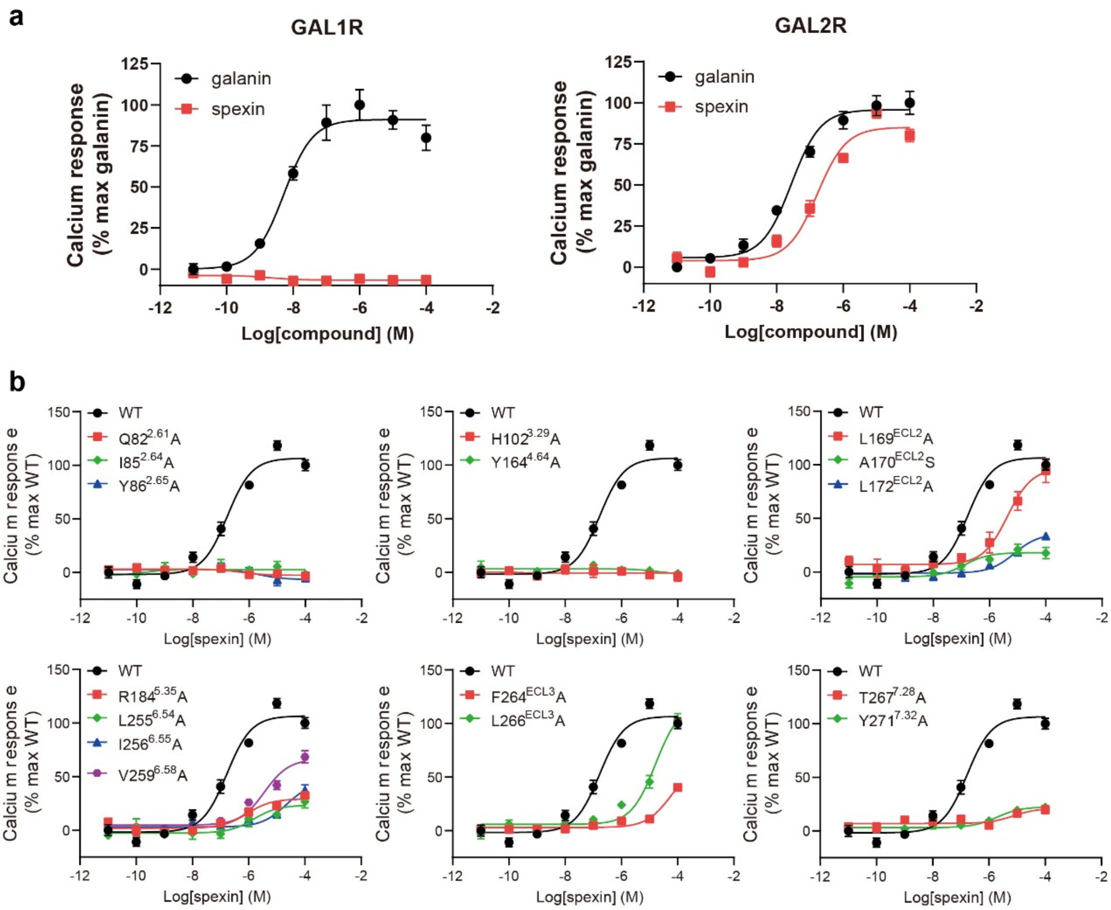

Supplementary Figure 9 | Spexin response curves on WT and mutant galanin receptors. **a,** Galanin and spexin response curves on WT GAL1R and GAL2R. **b,** Spexin response curves on WT and mutated GAL2R. WT or mutant GAL1R and GAL2R were transfected into HEK293/Gα_{16} cells and intracellular calcium signals were measured to reflect the activity of spexin. Each point represents mean ± S.E.M. from three independent experiments (n = 3). Source data are provided as a Source Data file.

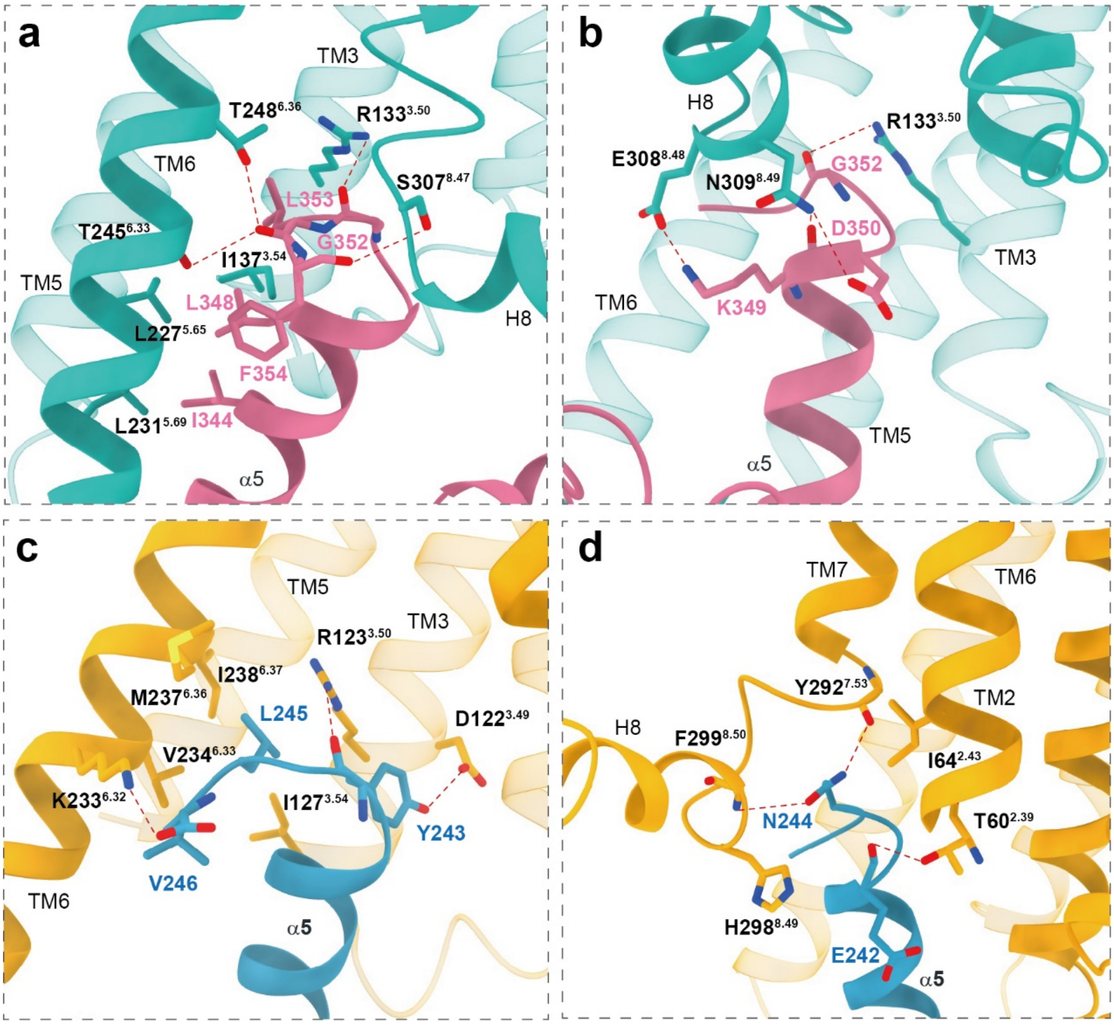

Supplementary Figure 10 | Detailed interactions between α5 helix of the Gα subunit and residues in cytoplasmic cavity of galanin receptors. a, b, Interactions between the α5 helix of the $G\alpha_i$ subunit and residues in GAL1R. **c, d,** Interactions between the α5 helix of the $G\alpha_q$ subunit and residues in GAL2R. Polar interactions are shown as red dashed lines.

Supplementary Table 1 | Cryo-EM data collection, model refinement, and validation statistics

	galanin-GAL1R-G_i-ScFv16	galanin-GAL2R-G_q-Nb35
**Data collection and processing**		
Magnification	4,9310	4,9310
Voltage (kV)	300	300
Electron exposure (e^-/Å^2)	64	64
Defocus range (μm)	-0.5 ~ -2.5	-0.5 ~ -2.5
Pixel size (Å)	1.014	1.014
Symmetry imposed	C1	C1
Initial particle projections (no.)	2,036,106	1,835,716
Final particle projections (no.)	340,033	255,766
Map resolution (Å)	2.7	2.6
FSC threshold	0.143	0.143
Map resolution range (Å)	2.2-4.0	2.1-4.0
**Refinement**		
Initial model used (PDB code)	7E32	6WHA, 6NBF
Model resolution (Å)	2.9	2.8
FSC threshold	0.5	0.5
Model resolution range (Å)	2.2-5.0	2.1-5.0
Map sharpening mothod	Relion	Relion
Model composition		
Non-hydrogen atoms	9209	8300
Protein residues	1160	1050
lipid	5	2
B factors (Å^2)		
Protein	88.98	81.62
lipid	109.09	95.13
R.m.s. deviations		
Bond lengths (Å)	0.002	0.002
Bond angles (°)	0.441	0.467
Validation		
MolProbity score	1.26	1.37
Clashscore	4.99	6.69
Rotamer outliers (%)	0.40	0.45
Ramachandran plot		
Favored (%)	99.04	99.23
Allowed (%)	0.96	0.77
Disallowed (%)	0.00	0.00

Supplementary Table 2 | Binding and function of galanin mutants on WT galanin receptors

Galanin Mutants	GAL1R				GAL2R			
	Binding assay		Calcium assay		Binding assay		Calcium assay	
	K_i (nM) [a]	I_{max} (%) [a,b]	EC_{50} (nM) [a]	E_{max} (%) [a,c]	K_i (nM) [a]	I_{max} (%) [a,c]	EC_{50} (nM) [a]	E_{max} (%) [a,b]
Galanin	10.7±0.3	100±0.5	14.2±2.8	100±3.0	23.7±1.2	100±0.7	12.7±3.0	100±3.0
Gal (1-16)	19.4±2.1	98.2±0.6*	15.3±6.7	95.5±5.2	19.4±2.1	101±0.9	16.2±3.7	103±2.5
Gal (1-13)	356±28*	96.2±0.5*	307±20*	88.1±4.1*	23.5±9.2	98.5±0.7	32.1±18	101±0.9
Gal (2-16)	>10000	37.5±3.0***	3316±83**	79.8±5.3	49.9±16	99.5±0.9	24.9±13	99.2±6.8
Gal (1-16, Y9A)	2475±390*	75.9±1.6***	>10000	14.4±4.0***	>100000	2.5±2.9***	>10000	32.8±4.9***

[a] Data shown are means ± S.E.M. from at least three independent experiments. *P<0.01; **P<0.001 and ***P<0.0001 by one-way ANOVA followed by Dunnett's multiple comparisons test, gal (1-16) and gal (1-13) compared with galanin, gal (2-16) and gal (1-16, Y9A) compared with gal (1-16).

[b] I_{max} is the inhibition of galanin-A2 (50 nM) binding to the receptors by ligands at 100 μM, normalized to the inhibition of galanin-A2 (50 nM) binding to the receptors by galanin at 100 μM.

[c] E_{max} is the response of ligands at 100 μM, normalized to the response of galanin at 100 μM.

Supplementary Table 3 | Binding of galanin-A2 and Function of galanin on GAL1R mutants

GAL1R Mutants	Ligand-binding assay		Calcium assay	
	K_d (nM) [a]	B_{max} (%) [a,b]	EC_{50} (nM) [a]	E_{max} (%) [a,c]
WT	22.19±0.89	100 ±1.2	15.2±3.7	100±0.8
E32$^{1.31}$R	>500	55.0 ±2.2**	251±58*	89.1±3.6*
Q92$^{2.61}$A	>500	35.7 ±5.0**	283±65*	85.8±5.7
V95$^{2.64}$A	UD [d]	UD	1696±494*	91.6±1.7*
Y96$^{2.65}$A	UD	UD	22780±301***	75.2±4.1**
H112$^{3.29}$A	59.76±3.0**	92.8 ±0.8*	42.4±7.7*	100±3.8
Q174$^{4.64}$A	351.0±40*	130±7.2	76.4±13*	90.2±10.4
F177^{ECL2}A	305.0±34*	104 ±7.7	58.3±4.2**	133±9.2*
F186^{ECL2}A	UD	UD	1405±189**	82.8±5.7*
W188^{ECL2}A	UD	UD	1489±107***	89.3±5.2
K197$^{5.35}$A	159.2±13**	111 ±3.0	47.8±23	72.1±5.9**
I266$^{6.54}$A	244.9±18**	108 ±5.2	80.4±6.1***	97.4±1.1
A270$^{6.58}$S	167.7±8.7**	106 ±3.6	41.8±7.9*	107±9.3
F275^{ECL3}A	UD	UD	3261±607**	104±6.9
L277^{ECL3}A	UD	UD	108±19**	105±2.8
F282$^{7.32}$A	UD	UD	>10000	36.1±14.7*
H263$^{6.51}$A	212.7±18*	119 ±4.6	163±20*	75.5±5.9*
H289$^{7.39}$A	135.5±5.8**	64.6 ±0.7***	92.3±29	31.4±8.8**
R285$^{7.35}$A	158.7±14*	99.2 ±4.9	69.8±5.5**	19±4.6***
W260$^{6.48}$A	51.2±7.3	42.9±1.6***	NR[e]	NR

[a] Data shown are means ± S.E.M. from at least three independent experiments. *P<0.01; **P<0.001 and ***P<0.0001 by one-way ANOVA followed by Dunnett's multiple comparisons test, compared with WT.

[b] B_{max} is the specific binding of 500 nM galanin-A2 on GAL1R mutant compared to WT receptor.

[c] E_{max} is the response of 100 μM galanin on GAL1R mutant compared to WT receptor.

[d] UD, undetectable.

[e] NR, no response, refers to response < 10% compared to WT receptor (galanin at 100 μM).

Supplementary Table 4 | Binding of galanin-A2 and Function of galanin on GAL2R mutants

GAL2R Mutants	Ligand-binding assay		Calcium assay	
	K_d (nM) [a]	B_{max} (%) [a,b]	EC_{50} (nM) [a]	E_{max} (%) [a,c]
WT	21.69±1.8	100±2.5	42.5±11	100±4.1
Q82$^{2.61}$A	>500	16.1±5.4**	3997±519**	36.7±2.7***
I85$^{2.64}$A	UD[d]	UD	3674±1168*	77.7±7.6
Y86$^{2.65}$A	UD	UD	2204±593*	23.2±9.7**
H102$^{3.29}$A	UD	UD	NR[e]	NR
Y164$^{4.64}$A	UD	UD	NR	NR
L169^{ECL2}A	86.17±10*	74.2±3.4*	66.2±22	69.1±3.9**
A170^{ECL2}S	33.53±1.0	105±2.1	10.2±1.2*	74.5±4.2*
L172^{ECL2}A	51.56±5.4*	78.7±2.0*	62.0±4.9	112±6.2
R184$^{5.35}$A	286.2±14**	93.4±3.2	104±24	40.3±2.2***
L255$^{6.54}$A	55.11±3.9**	34.0±1.3***	153±81	22.5±4.3***
I256$^{6.55}$A	220.6±15**	139±4.4*	169±81	46.4±15.5*
V259$^{6.58}$A	179.7±15*	109±5.8	257±26**	101±1.7
F264^{ECL3}A	>500	16.7 ±1.5***	2581±536**	64.9±9.5*
L266^{ECL3}A	UD	UD	16515±6062*	93.0±3.1
T267$^{7.28}$A	229.2±36*	49.3±4.3**	2245.7±548*	48.3±7.6**
Y271$^{7.32}$A	>500	10.2±3.4***	721±133**	57.6±4.3**
H252$^{6.51}$A	245.8±47	71.6±8.6	NR	NR
H278$^{7.39}$A	UD	UD	127±18*	22.8±4.3***
R274$^{7.35}$A	552.6±135	114±21	171±17**	68.3±0.6**
W249$^{6.48}$A	23.28±1.2	140±1.7**	NR	NR

[a] Data shown are means ± S.E.M. from at least three independent experiments. *P<0.01; **P<0.001 and ***P<0.0001 by one-way ANOVA followed by Dunnett's multiple comparisons test, compared with WT.

[b] B_{max} is the specific binding of 500 nM galanin-A2 on GAL2R mutant compared to WT receptor.

[c] E_{max} is the response of 100 μM galanin on GAL2R mutant compared to WT receptor.

[d] UD, undetectable.

[e] NR, no response, refers to response < 10% compared to WT receptor (galanin at 100 μM).

Supplementary Table 5 | Function of spexin on GAL2R mutants

GAL2R Mutants	Calcium assay	
	EC_{50} (nM) [a]	E_{max} (%) [a,b]
WT	195±71	100±4.9
Q82$^{2.61}$A	NR[c]	NR
I85$^{2.64}$A	NR	NR
Y86$^{2.65}$A	NR	NR
H102$^{3.29}$A	NR	NR
Y164$^{4.64}$A	NR	NR
L169^{ECL2}A	4712±1560*	94.58±11.1
A170^{ECL2}S	132±19	17.66±5.3***
L172^{ECL2}A	8665±279*	33.56±1.2***
R184$^{5.35}$A	3975±3300	32.36±1.6***
L255$^{6.54}$A	1770±1240	26.65±5.9***
I256$^{6.55}$A	679±140*	67.24±0.5**
V259$^{6.58}$A	4700±1691	68.4±5.7*
F264^{ECL3}A	>100000	40.26±1.3***
L266^{ECL3}A	17470±3950*	103.14±6.2
T267$^{7.28}$A	3998±3274	19.9±3.4***
Y271$^{7.32}$A	2763±1615	22.23±2.2***

[a] Data shown are means ± S.E.M. from at least three independent experiments. *P<0.01; **P<0.001 and ***P<0.0001 by one-way ANOVA followed by Dunnett's multiple comparisons test, compared with WT.

[b] E_{max} is the response of 100 μM spexin on GAL2R mutant compared to WT receptor.

[c] NR, no response, refers to response < 10% compared to WT receptor (spexin at 100 μM).
