# Supplementary material for: Molecular basis for allosteric agonism and G protein subtype selectivity of galanin receptors
Source: Nat Commun. 2022 Mar 15;13:1364. doi: 10.1038/s41467-022-29072-3 (PMC8924211; doi:10.1038/s41467-022-29072-3)
Supplement: Supplementary file 2 — Reporting Summary [file 41467_2022_29072_MOESM2_ESM.pdf]

nature research

Corresponding author(s): Yi Jiang
Xin Xie
Yan Zhang
H. Eric Xu

Last updated by author(s): 2/12/2022

Reporting Summary

Nature Research wishes to improve the reproducibility of the work that we publish. This form provides structure for consistency and transparency in reporting. For further information on Nature Research policies, see our Editorial Policies and the Editorial Policy Checklist.

Statistics

For all statistical analyses, confirm that the following items are present in the figure legend, table legend, main text, or Methods section.

n/a	Confirmed	
☐	☒	The exact sample size (*n*) for each experimental group/condition, given as a discrete number and unit of measurement
☐	☒	A statement on whether measurements were taken from distinct samples or whether the same sample was measured repeatedly
☐	☒	The statistical test(s) used AND whether they are one- or two-sided<br>*Only common tests should be described solely by name; describe more complex techniques in the Methods section.*
☒	☐	A description of all covariates tested
☒	☐	A description of any assumptions or corrections, such as tests of normality and adjustment for multiple comparisons
☐	☒	A full description of the statistical parameters including central tendency (e.g. means) or other basic estimates (e.g. regression coefficient) AND variation (e.g. standard deviation) or associated estimates of uncertainty (e.g. confidence intervals)
☐	☒	For null hypothesis testing, the test statistic (e.g. *F*, *t*, *r*) with confidence intervals, effect sizes, degrees of freedom and *P* value noted<br>*Give P values as exact values whenever suitable.*
☒	☐	For Bayesian analysis, information on the choice of priors and Markov chain Monte Carlo settings
☒	☐	For hierarchical and complex designs, identification of the appropriate level for tests and full reporting of outcomes
☒	☐	Estimates of effect sizes (e.g. Cohen's *d*, Pearson's *r*), indicating how they were calculated

Our web collection on statistics for biologists contains articles on many of the points above.

Software and code

Policy information about availability of computer code

Data collection	Automated data collection on the Titan Krios using serialEM 3.8.
Data analysis	Relion 3.0, MotionCor2.1, Gctf 1.18, UCSF Chimera v1.13.1, Coot 0.9 EL, Rosetta 3.13, Phenix 1.18.2, Prism8.

For manuscripts utilizing custom algorithms or software that are central to the research but not yet described in published literature, software must be made available to editors and reviewers. We strongly encourage code deposition in a community repository (e.g. GitHub). See the Nature Research guidelines for submitting code & software for further information.

Data

Policy information about availability of data

All manuscripts must include a data availability statement. This statement should provide the following information, where applicable:

- Accession codes, unique identifiers, or web links for publicly available datasets
- A list of figures that have associated raw data
- A description of any restrictions on data availability

The atomic coordinates and the electron microscopy maps have been deposited in the Protein Data Bank (PDB) under accession number 7WQ3 and 7WQ4 and Electron Microscopy Data Bank (EMDB) accession number EMD-32698 and EMD-32699 for the galanin-GAL1R-Gi-ScFv16 and the galanin-GAL2R-Gq-Nb35 complex, respectively. Source data are provided with this paper.

Field-specific reporting

Please select the one below that is the best fit for your research. If you are not sure, read the appropriate sections before making your selection.

☒ Life sciences ☐ Behavioural & social sciences ☐ Ecological, evolutionary & environmental sciences

For a reference copy of the document with all sections, see nature.com/documents/nr-reporting-summary-flat.pdf

Life sciences study design

All studies must disclose on these points even when the disclosure is negative.

Sample size	Sample sizes were not predetermined by statistical methods. For cryo-EM data, images were collected until the resolution and 3D reconstruction converges. For all the functional assay, we use sample size at least of three independent experiments, commonly exploited by researchers in this field.
Data exclusions	No data were excluded from the analysis.
Replication	Experimental findings were reliably reproduced within one month.
Randomization	Randomization was not relevant to this study, as the data were collected automatically and did not involve choosing.
Blinding	Blinding is not necessary or valid for the purposes of structural determination. For cryo-EM study, imaging data were collected automatically. For functional analysis, blinding was not necessary by reason of the quantitative nature of the experiment.

Reporting for specific materials, systems and methods

We require information from authors about some types of materials, experimental systems and methods used in many studies. Here, indicate whether each material, system or method listed is relevant to your study. If you are not sure if a list item applies to your research, read the appropriate section before selecting a response.

Materials & experimental systems

n/a	Involved in the study	
☒	☐	Antibodies
☐	☒	Eukaryotic cell lines
☒	☐	Palaeontology and archaeology
☒	☐	Animals and other organisms
☒	☐	Human research participants
☒	☐	Clinical data
☒	☐	Dual use research of concern

Methods

n/a	Involved in the study	
☒	☐	ChIP-seq
☒	☐	Flow cytometry
☒	☐	MRI-based neuroimaging

Eukaryotic cell lines

Policy information about cell lines

Cell line source(s)	Sf9 (Expression Systems, Cat#94-001F)<br>HEK 293 (Cell Bank at the Chinese Academy of Sciences)
Authentication	Used as expression stains only, independent verification after purchase not required.
Mycoplasma contamination	Cell lines were tested and free from mycoplasma contamination.
Commonly misidentified lines<br>(See ICLAC register)	No commonly misidentified cell lines were used.
